# Supplementary material for: The genome sequence of a leafhopper, Allygus modestus Scott, 1876
Source: Wellcome Open Res. 2025 Jan 14;10:9. [Version 1] doi: 10.12688/wellcomeopenres.23451.1 (PMC11775446; doi:10.12688/wellcomeopenres.23451.1)
Supplement: Peer review history

# Reviewer report: Reviewer response for version 1

**DOI:** 10.21956/wellcomeopenres.25861.r117270

**Published:** 2025-02-03

**Authors:** Zheng Li

**Affiliations:** 1 Miami University Department of Biology, Oxford, Ohio, USA

This paper provides a chromosome-level genome assembly of a leafhopper, *Allygus modestus*. This genome will be a wonderful resource for many insect researchers. The quality of the genome assembly meets the standard of other insect genomes from the Darwin tree of life project.  

 I have some minor comments and suggestions: 

1. ‘Allygus mixtus’ should be italicized.
2. It is unclear how the X chromosome is confirmed. I assume it is probably based on the read depth. It will be helpful to mention that in the manuscript.
3. It will be better to confirm the chromosome number through karyotyping. 

Are sufficient details of methods and materials provided to allow replication by others?

Yes

Is the rationale for creating the dataset(s) clearly described?

Yes

Are the datasets clearly presented in a useable and accessible format?

Yes

Are the protocols appropriate and is the work technically sound?

Yes

Reviewer Expertise:

genome evolution

I confirm that I have read this submission and believe that I have an appropriate level of expertise to confirm that it is of an acceptable scientific standard.

# Reviewer report: Reviewer response for version 1

**DOI:** 10.21956/wellcomeopenres.25861.r117274

**Published:** 2025-01-28

**Authors:** Yiyuan Li

**Affiliations:** 1 Ningbo University, Zhejiang, China

In this paper, the authors assembled the genome of *Allygus modestus*. The genome is of high quality with six chromosomes and a X chromosome assembled. The authors used a single male individual for both HiFi and HiC sequencing helped identify the X chromosome and reduce the heterozygosity from pooling multiple individuals. Overall, I think the authors did a great job showing that it is possible to sequence the genome of an individual leafhopper. I only have a few minor comments:

 (1) It would be useful to add chromosome IDs to Figure 5.

 (2) There is a red cross on Chr 2 of Figure 5. Do the red lines represent the boundaries of scaffolds? As there are also grey lines representing the boundaries of different chromosomes, it would be helpful to add details about the red/grey lines in the figure legend.

Are sufficient details of methods and materials provided to allow replication by others?

Yes

Is the rationale for creating the dataset(s) clearly described?

Yes

Are the datasets clearly presented in a useable and accessible format?

Yes

Are the protocols appropriate and is the work technically sound?

Yes

Reviewer Expertise:

Insect genomics, comparative genomics, evolutionary biology

I confirm that I have read this submission and believe that I have an appropriate level of expertise to confirm that it is of an acceptable scientific standard.

# Reviewer report: Reviewer response for version 1

**DOI:** 10.21956/wellcomeopenres.25861.r117278

**Published:** 2025-01-24

**Authors:** Roberto Biello

**Affiliations:** 1 John Innes Centre Department of Crop Genetics, Norwich, England, UK; 2 University of Ferrara, Ferrara, Italy

This manuscript presents the genome sequence of a Cicadellidae species, *Allygus modestus*, that is a group with not many genomics resources available. Considering that Cicadellidae are mainly plant-feeding sap feeders, can act as vectors transmitting plant pathogens, and can be significant agricultural pests, new resources, like this study, can improve our understanding of this important group of Hemiptera. 

 The methods are well described, and the results are pertinent. All raw and analysed datasets are deposited in publicly available repositories. The genome quality looks excellent.

 I only have minor comments:

 -    In the sentence, “This species, along with the similar Allygus mixtus (Fabricius, 1794),” the name *Allygus mixtus* should be italicized.

 -    In the introduction, the authors should clarify the current knowledge about this species, specifically regarding its geographic distribution.

 -    The authors should explain why a male was sequenced instead of a female, considering that females should have two X chromosomes.

 -    It would be helpful if the authors could cite studies where the karyotype of *A. modestus* was investigated through other methods, if available.

 -    Since the authors extracted DNA from the entire organism, I would expect that they also sequenced and assembled the genomes of symbionts (e.g. *Sulcia muelleri*) although this was not mentioned.

Are sufficient details of methods and materials provided to allow replication by others?

Yes

Is the rationale for creating the dataset(s) clearly described?

Yes

Are the datasets clearly presented in a useable and accessible format?

Yes

Are the protocols appropriate and is the work technically sound?

Yes

Reviewer Expertise:

My area of research focuses on insect evolution.

I confirm that I have read this submission and believe that I have an appropriate level of expertise to confirm that it is of an acceptable scientific standard.
